# Assessing Cognitive Impairment

**Published:** 1995

**Authors:** Sara Jo Nixon

**Affiliations:** Sara Jo Nixon, Ph.D., is the director of the Cognitive Studies Laboratory, Oklahoma Center for Alcohol and Drug Related Studies, and an associate professor in the Department of Psychiatry and Behavioral Sciences, University of Oklahoma Health Sciences Center, Oklahoma City, Oklahoma

**Keywords:** cognitive process, AOD impairment, neuropsychological assessment, neuroimaging, acute AODE (alcohol and other drug effects), chronic AODE

## Abstract

A plethora of tools and methods are being used to evaluate alcohol’s effects on cognitive functioning. Neuropsychological tests focus on identifying the brain regions affected by alcohol, whereas neurocognitive approaches attempt to distinguish impaired cognitive processes. These approaches are supplemented by neurophysiological and neuroimaging tools. Studies using all these instruments have characterized alcohol’s effects on cognitive functioning after both acute and chronic alcohol consumption. However, there are limitations to the generalizability of these findings because the subjects in the vast majority of studies do not adequately represent all subgroups of the general population.

Chronic alcohol abuse frequently is accompanied by significant impairment in mental abilities (Parsons and Nixon 1993; Knight and Longmore 1994), and even acute alcohol consumption (i.e., a single drinking episode) temporarily can affect the drinker’s cognitive functioning. However, the exact nature of the cognitive impairment caused by alcohol (i.e., the mechanisms underlying impairment) remains confusing and controversial. Some of this confusion stems from a lack of adequate description by researchers of how cognitive functioning has been assessed. Such vague descriptions are particularly apparent in review articles and in reports in the popular media. A large number of different tests and methods are used to evaluate cognitive functioning in alcohol studies. Failure to characterize the approach used in the studies limits the interpretation and application of current findings.

To illustrate how cognitive functions and cognitive impairment can be assessed, this article reviews some of the methods used to evaluate alcohol’s impact on cognitive performance. These methods include neuropsychological and neurocognitive tests as well as neuroimaging techniques. Although most of these techniques primarily are used to analyze cognitive impairment resulting from chronic alcohol abuse or dependence, the results of studies assessing the cognitive consequences of acute alcohol consumption also are mentioned. The article concludes with a discussion of factors that may limit the general applicability of current research findings, specifically age and gender biases in the choice of study subjects.

## Neuropsychological and Neurocognitive Assessment

Much of the work regarding the effects of chronic alcohol consumption on mental processes has focused on identifying specific neuropsychological domains—and the brain regions associated with them—that are affected by alcohol. These empirically determined functional domains describe areas of cognitive or psychological functioning, such as abstract thinking and problem-solving or perceptual-motor skills.

In early studies of alcohol’s effects, cognitive test batteries generally evaluated overall intelligence and neuropsychological functioning. Researchers since have refined these tests to assess more specific aspects of intelligence or neuropsychological functioning. An exhaustive review of all the instruments currently in use is beyond the scope of this article. Instead, a selection of these tools is presented in table 1. These tests often tap skills from more than one neuropsychological domain and thus reflect more closely the complex processes involved in cognitive functions.

Many of the tests described in table 1 are capable of detecting rather subtle cognitive dysfunction. The relevance of such subtle impairment is unclear. Although detoxified alcoholics typically achieve significantly lower scores on these tests than do control subjects of equivalent age, educational level, and socioeconomic status, their level of dysfunction cannot necessarily be considered “clinically impaired.” Some critics have argued that the sensitivity of these tests has been achieved at the cost of relevancy (for a discussion, see Nixon 1993). In other words, a subject who performs poorly on these carefully developed laboratory tests may be able to function quite well in the “real” world. In response to this concern, new assessment tools increasingly focus on tasks relevant to real-life situations. As illustrated in table 2, these tasks assess performance in several cognitive skill areas.

### The Neurocognitive Assessment Approach

In recent years, a new approach adopted from the field of neurocognitive science has been applied to studies of chronic alcohol effects. This approach focuses on the specific processes underlying cognitive functioning (e.g., perceiving, learning, and remembering information). Although the specific performance requirements are similar for both the neurocognitive and the traditional neuropsychological tests, the variables being measured and analyzed are different.

For example, in both approaches, subjects may be asked to memorize a story, associate pairs of items, or identify a target. The neuropsychological approach might focus on determining whether skills associated with right hemisphere function (e.g., visuospatial skills, such as replicating designs or geometric figures) or with left hemisphere function (e.g., verbal skills, such as identifying meaningful words) are impaired after chronic alcohol abuse. In contrast, the neurocognitive approach might assess alcohol’s differential effects on processes such as the perception, encoding (i.e., processing of information in the brain), or retrieval of information.

Because of their different focuses, the two approaches also differ in their clinical significance. For example, the neuropsychological approach can locate an impaired brain region (e.g., damage to the right or left hemisphere) based on the functional impairment observed. Similarly, neuropsychological tests can identify brain regions and their associated functional domains that have been spared from damage. The neurocognitive approach, on the other hand, by identifying underlying cognitive processes or mechanisms, may help develop treatment strategies attempting to rehabilitate these *specific* processes.

The increasing interaction of basic neuropsychological research and process-oriented neurocognitive research represents a new phase in the study of alcohol-related cognitive impairment. Some studies incorporate both neuropsychological and neurocognitive techniques. Although this combined approach requires a higher time commitment both for participants and for researchers, it creates an extensive knowledge base regarding brain/behavior relations that may help improve the understanding and treatment of alcohol-induced cognitive dysfunction.

## Neuropsychological Consequences of Chronic Alcohol Abuse

The findings regarding cognitive deficits following chronic alcohol abuse vary considerably. However, most neuropsychological studies indicate that detoxified alcoholics frequently demonstrate long-lasting deficits in four domains: abstract thinking and problem-solving skills, verbal skills and/or memory, perceptual-motor skills (e.g., putting pegs in a pegboard or finger dexterity), and visuospatial skills (Glenn et al. 1993; Tivis et al. 1995). (A discussion of the association of these domains with specific brain regions is outside the scope of this article. For reviews, see Lezak 1983; Kolb and Wishaw 1985; and Parsons et al. 1987.)

The neuropsychological domains appear to be differentially sensitive to the effects of chronic alcohol consumption. Whereas abstract thinking, perceptual-motor skills, and visuospatial skills almost always are affected by chronic alcohol abuse, alcohol-related deficits in verbal skills appear less frequently (Parsons 1987). The reason for this pattern is not entirely clear. It could be related to the fact that typical verbal tasks often involve over-practiced skills (i.e., skills performed many times in everyday situations), which are relatively impervious to decline. This conclusion is supported by studies that detect significant deficits in verbal skills after chronic alcohol abuse when difficult verbal tasks are used (Parsons 1987). However, additional work is needed to clarify the nature of these verbal deficits.

## Neurophysiology and Neuroimaging

In addition to performance-based neuropsychological and neurocognitive tests, neuroimaging techniques also are used to assess alcohol-related cognitive impairment. These techniques include electroencephalography (EEG), magnetic resonance imaging (MRI), positron emission tomography (PET), single photon emission tomography (SPECT) and computerized tomography (CT). (For a review of these and other techniques, see Zakhari and Witt 1992.) Although all these techniques can detect neurophysiological changes associated with chronic alcohol consumption, EEG’s are used most frequently. This is due in part to the technical aspects of data collection and interpretation, the relatively low cost, and the ready availability of EEG’s. In the study of cognitive processes, specific EEG components called event-related potentials (ERP’s) often are used. ERP’s are changes in the brain’s electrical activity in response to the presentation of discrete stimuli. ERP’s consist of several components (“peaks” and “valleys”) that occur at different times after stimulus presentation and that appear to be related to specific aspects of cognitive functioning. (For additional information on these components and how they are affected by alcohol, see the article by Porjesz and Begleiter, pp. 108–112.)

Chronic alcohol consumption is frequently associated with alterations in the P300 component, a positive component (i.e., peak) of the ERP occurring approximately 300 milliseconds after the presentation of a relevant, but rare, stimulus. In a prototypical experiment called the odd-ball paradigm, subjects must attend to a specific infrequent stimulus (e.g., count the number of occurrences) while ignoring another more frequently occurring stimulus. For example, subjects observing a series of red and green dots appearing in rapid succession on a computer screen, with the green dots outnumbering the red ones, are asked to count the “rare” red dots. The occurrence of the rare yet relevant red dots among the irrelevant green dots elicits a P300 ERP response.

The P300 has been associated with target identification and the memory updating system (i.e., recognizing and remembering a stimulus) (Coles et al. 1990). Current research generally indicates that alcoholics produce delayed and/or smaller P300 peaks than do control subjects (for a review, see Porjesz and Begleiter 1993). The implications of this finding have not been fully developed; current data indicate only a modest correlation between neurophysiological aberrations and cognitive performance as measured by behavioral tests, such as those in tables 1 and 2. More research directed at understanding this inconsistency is needed to elucidate the effects of chronic alcohol consumption on the relationship between brain neurophysiology and behavior.

## Cognitive Effects of Acute Alcohol Administration

Although the types of tasks used to assess acute alcohol effects often are similar to tasks used to assess chronic alcohol effects, the primary focus of the two types of studies has been different. Specifically, contrary to the studies of long-term alcohol effects on cognition, studies of acute effects have tended to focus on tasks that assess performance of functions relevant to driving and other practical skills. Thus, many of these studies have used an information-processing approach with particular interest in changes in reaction time and response accuracy under various conditions of alcohol exposure.

There have been literally thousands of studies conducted on the effects of acute alcohol administration. Several reviews, such as the ones described below, attempt to integrate this vast literature. In addition to summarizing the general findings, these reviews illustrate the broad range of variables and techniques used to assess alcohol’s effects on cognitive functioning.

Moskowitz and Robinson (1988) reviewed 158 studies conducted between 1940 and 1985, considering nine performance measures: reaction time (i.e., responding to a specific stimulus), tracking (i.e., following the movement of an object on a computer screen), vigilance (i.e., responding to an infrequent, relevant stimulus against a background of frequent, irrelevant stimuli), divided attention (i.e., performing two tasks at the same time), visual function, information processing, perception, psychomotor skills, and driving. The authors concluded that divided attention tasks provided the most sensitive measure of impairment. Sixty percent of the studies reviewed detected impairment at blood alcohol concentrations (BAC’s) at or below 0.05 percent^1^ with divided attention tasks. Performance of the other tasks was not impaired to the same extent.

More recently, Holloway (1994) summarized 148 studies conducted between 1985 and 1993. This review focused on studies addressing the effects of a range of low doses of alcohol that are relevant for social drinkers. Instead of focusing only on performance tasks, especially those obviously related to driving and other high-risk behaviors, Holloway also considered the effects of alcohol on subjective measures (e.g., negative effects, such as feeling intoxicated, and positive effects, such as feeling euphoric) and cognitive measures, such as memory. Some of the findings from this review, as observed across studies, are summarized as follows:

- People experience alcohol’s subjective intoxicating effects at lower BAC’s than alcohol-induced performance impairment.
- Whereas a linear relationship exists between BAC and performance impairment over a wide range of BAC’s (i.e., the higher the BAC, the stronger the performance impairment), there appears to be a threshold BAC below which people do not experience subjective intoxication.
- The effects of alcohol are greater on tasks or processes demanding attention or effort (i.e., “controlled” processes) than on tasks or processes making few attentional demands (i.e., “automatic” processes).
- Seventy to 80 percent of the studies found that BAC’s at or below 0.04 percent had significant effects on intoxication ratings and on the performance of controlled tasks.
- Characteristics of the study subjects, such as expectancy^2^ and tolerance,^3^ as well as contextual parameters (e.g., time of day or social environment), may influence sensitivity to the effects of alcohol.

Rather than consider alcohol’s effects only on specific behavioral tasks, some researchers have suggested that acute alcohol administration has a global effect on cognitive functioning. For example, Steele and Josephs (1990) have suggested that alcohol produces a “myopic” effect, allowing the drinker to focus attention only on the most salient aspects of any given situation and disregarding the significance of other aspects. For example, a person who has been drinking may only focus on and react to the annoying or provocative nature of an acquaintance, rather than considering the implications of reacting in anger.

Although alcohol-related myopia is unlikely to account for alcohol’s effects on behavior in all contexts, other studies support this interpretation. For example, Zeichner and colleagues (1993) examined how much time intoxicated subjects spent reading adjectives that described positive (e.g., bright or polite) or negative (e.g., stubborn or foolish) personality traits of the subjects themselves (i.e., were salient) or of another person (i.e., were nonsalient). Consistent with the myopia hypothesis, subjects spent significantly more time attending to personally salient, negative traits than they did to other types of traits. However, more work is needed to determine if this pattern is observed in situations more similar to everyday life.

## Limitations of Current Research Findings

Although numerous studies have analyzed alcohol’s effects on cognitive functioning and performance, their findings may not apply to all members of the population to the same extent. For example, the vast majority of studies include only young, healthy men and thus may not reflect alcohol’s effects on older people or women. This bias in the selection of study subjects may be the result of concerns about undetected pregnancy in women or the lack of appropriate control subjects among older people.

Few studies have analyzed gender differences in the consequences of acute alcohol effects on cognitive functioning. Niaura and colleagues (1987) examined the effects of an acute alcohol dose on psychomotor performance, pharmacokinetic response, and cognitive impairment in men and women. This study found a stronger effect on women only in the development of acute tolerance to alcohol-induced memory impairment. Other reports have suggested more wide-ranging gender differences in the effects of acute alcohol doses (Sutker et al. 1982; Wait et al. 1982). However, current research suggests that when the alcohol dose is adjusted for gender differences in body fat—women generally have a higher proportion of body fat than men—at least some of these differences may be eliminated (Nicholson et al. 1992).

Existing data indicate similar patterns of cognitive dysfunction in male and female alcoholics (i.e., after chronic alcohol consumption). This is remarkable because female alcoholics consistently report fewer years of drinking and/or a lesser quantity per drinking occasion than male alcoholics (for reviews, see Glenn 1993; National Institute on Alcohol Abuse and Alcoholism 1990). Based on these findings, it has been suggested that women have a “telescoped,” or accelerated, progression of the negative consequences of alcohol consumption (Glenn 1993).

## Summary

Numerous diagnostic instruments are available to assess alcohol-related changes in cognitive functioning and to detect subtle and specific impairment. Based on the performance of specific tasks, neuropsychological and neurocognitive tests can help identify both the brain structures and the cognitive processes affected by alcohol consumption. These findings may contribute to the development of treatment approaches appropriate for patients with different kinds of cognitive impairment. Furthermore, by revealing information about cognitive impairment, these findings indirectly may stimulate new approaches to the study of the brains of healthy persons. However, to maximize the benefits from these tools, researchers must carefully select the test best suited to answer their specific research question and document how cognitive functioning has been assessed.

The testing instruments have been used in many studies and have provided insight into the consequences of chronic and acute alcohol consumption. However, there are limitations to this work. Foremost is the fact that most of the studies have used primarily healthy young men as subjects. Relatively few studies specifically have assessed the neuropsychological, neurocognitive, and neurophysiological consequences of alcohol consumption in other population subgroups. Preliminary findings in older people and women underscore the necessity of expanding this line of research to understand fully alcohol’s effects on cognitive functioning.

## Figures and Tables

**Table 1:** Tests Assessing Various Aspects of Cognitive Functioning

Overall Mental Assessment		

Test	Comment	Specifics
Wechsler Adult Intelligence Scale, Revised (WAIS-R; Wechsler 1987*a*)	Seldom administered in its entirety; subscales used to assess specific neuropsychological (NP) domains (see below).	11 subscales: 6 verbal scales and 5 performance scales. Provides measures of verbal IQ, performance IQ, and full-scale IQ.
Halstead-Reitan Battery (HRB; Russell et al. 1970)	Seldom administered in its entirety; subtests used to assess specific NP domains (see below).	Seven subtests addressing multiple NP domains, including frontal lobe function and right and left hemisphere function. Provides an “impairment index” as the ratio of failed versus normal tests.
Shipley Institute of Living Scale (Zachary 1986)	Provides “mental age” scores for vocabulary and abstracting skills. Allows estimate of overall conceptual quotient (i.e., a combination of vocabulary and abstracting skills) and WAIS-R IQ. Performance on the vocabulary test rarely is affected in chronic alcohol studies and often is used as a control measure.	40-item vocabulary test combined with a 20-item verbal problem-solving test. Both parts have items of varying difficulty.

**Learning and Memory: Verbal and Visuospatial**		

Wechsler Memory Scale (WMS; Wechsler 1945, 1987*b*)	Selected subtests generally are used for specific research questions (see below). Recent revisions of the scale may increase its use (for discussion, see Knight and Longmore 1994).	Seven subtests assessing general knowledge, mental control, short-term memory, verbal learning, and memory for stories and figural representations.
Russell’s Version WMS (Russell 1982)	Provides immediate and delayed (30 minutes) assessment, thus providing a means to determine memory function. Use of both subscales allows assessment of both left hemisphere (verbal memory) and right hemisphere (figural memory) function.	Uses two subscales of the WMS: memory for stories and memory for figural representations.
Luria Words Test (Verbal) (Luria 1976)	Ascertains acquisition patterns of information and differential memory. Uses common concrete nouns, thus reducing confounding issues of familiarity with the words or of abstraction.	Assesses acquisition and retention of 10 words over 3 time periods (2, 8, and 30 minutes).
Rey Auditory Verbal Learning Test (RAVLT; for information, see Lezak 1983)	Not commonly used in alcohol-related studies. Lack of a published manual may contribute to this omission.	Measures the acquisition and retention of multiple verbal lists.

**Conceptual Learning (Frontal Lobe Functioning)**		

Wisconsin Card Sorting Test (WCST; Heaton 1981)	Classic test of the ability to shift problem-solving strategies with minimal feedback. Successful subjects respond with a change in sorting strategy when told that a sort is “incorrect.” Computer assistance facilitates test administration and scoring.	Subject sorts cards into one of four piles based on color, form, or number. Primary DV’s^1^ are the number of categories completed, number of total errors, and number of perseverative errors (i.e., failure to change categories).
California Card Sorting Test (Delis et al. 1989; Beatty and Monson 1990, 1992; Beatty et al. 1993)	Task demands and possible DV’s are more extensive than in the WCST.	Determines the ability to generate, execute, and identify conceptual strategies. Stimuli consist of cards varying in shape, color, size, and verbal labels.
Conceptual Level Analogy Test (CLAT; Willner 1970, 1971)	Verbal problem-solving and abstracting test. Several studies using the CLAT have used abbreviated versions, reducing time requirements and providing items for test/retest protocols.	42 items addressing 6 levels of complexity, ranging from opposites (e.g., hot-cold, black-white) to 2-category contrasts (e.g., wolf-dog, hurricane-breeze).

**Perceptual-Motor Skills**		

Trail Making Test, Form A and B (Russell 1975)	Subtest of the HRB; overall, form B appears more sensitive to alcohol effects. Time to completion is the more frequently reported DV.	Form A: Subject connects numbered dots (number 1 to number 13) with a line. Form B: Subject connects alternating numbers and letters (1 to A to 2 to B, etc.) with a line. DV’s are time to completion and number of errors.
WAIS-R Digit Symbol Substitution Test (Wechsler 1987*a*)	A subtest of the WAIS frequently used in alcohol studies. Not a true memory test.	Subject copies the symbols assigned to digits 1 to 9 below each digit. The symbols always are available for reference. DV is the number of symbols correctly substituted in 90 seconds.
Grooved Pegboard Test (Sander et al. 1989)	Time to completion with the nondominant hand is more sensitive to alcohol effects.	Subject places round, grooved pegs in a grooved pegboard. Task is completed first with the dominant hand, then with the nondominant hand. DV is the time to completion.

**Visuospatial, Nonmemory Skills**		

Little Men Test (Acker and Acker 1982)	Computerized administration facilitates time and accuracy measurements.	A manikin holding a briefcase is shown in one of four positions (i.e., upright, inverted, facing toward the subject, or facing away from the subject). Subject identifies which hand is holding the briefcase. DV’s are reaction time and number of errors.
Mazes (Acker and Acker 1982)	Part of the same test battery as the Little Men Test. Computerized administration facilitates time and accuracy measurement.	Subject identifies matching but rotated mazes. DV’s are reaction time and number of errors.
WAIS-R Block Design (Wechsler 1987*a*)	Part of the WAIS-R test battery. Classic measure of visuospatial/perceptual-motor skills.	Subject reconstructs designs of red and white squares with a set of red and white blocks. DV’s are time to completion of individual designs, number of designs completed, and number of completion errors.

**Other Tests**		

Verbal Fluency (Thurstone 1938; Newcomb 1969)	Classic assessment of verbal production. Production has been studied for both phonetic and semantic tasks. Data from alcoholics are inconsistent. Some studies reveal significant effects; others do not (for review, see Hewett et al. 1991).	Subject produces as many words as possible beginning with a specific letter (phonetic) or belonging to a specific category (semantic) within a certain time.
Short-Term Memory (Sternberg 1966, 1975)	Various forms of Sternberg’s protocol for assessing short-term memory are common components in neurocognitive test batteries for alcoholics. Computerized administration facilitates reaction time and accuracy measurement.	A series of letters or numbers are presented individually at a rapid rate. An individual letter or number then is presented and the subject has to indicate whether it was presented before. Subject also has to recall the letters and numbers presented.

1 DV = Dependent variable, the measurement used in the test.

**Table 2:** Alternative Assessment Instruments

Test	Comment	Specifics
Plant Test (Erwin and Hunter 1984; Nixon and Parsons 1991)	Modeled from traditional cognitive development tasks, the test appeals to most participants. The task requires less than 5 minutes to administer.	Problem-solving task that requires the subject to identify and isolate relevant from irrelevant variables.
Adaptive Skills Battery (ASB; Jones and Lanyon 1981; Nixon et al. 1992)	Current data suggest that “typical” responses are more sensitive to alcohol effects. Test may require 30 to 45 minutes to administer. Standard scoring protocols are available.	Subject has to produce the “typical” response or the “best possible” response to 30 vignettes involving interpersonal relations.
Face-Name Learning (Becker et al. 1983; Schaeffer and Parsons 1987)	The multitrial presentation format allows measurement of learning curves as well as of final performance levels.	Subject must learn the correct names for individual faces. Stimuli are presented at a constant pace. Multiple sets of tests are performed.
Rivermead Behavioral Memory Test (RBMT; Wilson 1987; Wilson et al. 1985)	Has been applied primarily with Wernicke-Korsakoff patients.	11 subtests assess performance on items relevant to successful independent functioning (e.g., remembering a short route, hidden object, or appointment).
California Verbal Learning Test (CVLT; Delis et al. 1987, 1988)	The wide variety of dependent variables (i.e., measurements used in the test) makes the test appropriate for many alcohol-related questions.	Uses two different “shopping lists” to assess acquisition of verbal information. Dependent variables are rate of learning, use of strategies, accuracy, interference, order errors, persistence in errors, and confusion between lists.

## Footnotes

1 A BAC of 0.05 percent corresponds to 0.05 gram pure alcohol per 100 milliliters blood.

2 Expectancy means that a person expects certain effects on behavior after the consumption of alcohol; therefore, the person may display the expected behavioral effects whether or not alcohol actually is consumed.

3 Tolerance means that after continued alcohol consumption, a higher dose than before is required to elicit the same effects. Tolerance can develop between drinking sessions (chronic tolerance) or within one drinking session (acute tolerance). In the latter case, alcohol’s effects at a given BAC are higher during the rising phase (i.e., ascending limb) of the BAC curve than at the same BAC during the falling phase (i.e., descending limb) of the BAC curve.
